# Supplementary material for: Recognition and cleavage mechanism of intron-containing pre-tRNA by human TSEN endonuclease complex
Source: Nat Commun. 2023 Sep 28;14:6071. doi: 10.1038/s41467-023-41845-y (PMC10539383; doi:10.1038/s41467-023-41845-y)
Supplement: Supplementary file 1 — Supplementary Information [file 41467_2023_41845_MOESM1_ESM.pdf]

Supplementary Information

Recognition and cleavage mechanism of intron-containing pre-tRNA by human TSEN endonuclease complex

Ling Yuan[1], Yaoyao Han[2], Jiazheng Zhao[1], Yixiao Zhang[2#], Yadong Sun[1#]

[1]School of Life Science and Technology, ShanghaiTech University, Shanghai, China

[2]Interdisciplinary Research Center on Biology and Chemistry, Shanghai Institute of Organic Chemistry, Chinese Academy of Sciences, China.

# Corresponding authors. Email: sunyd1@shanghaitech.edu.cn (Y.S.); yzhang@mail.sioc.ac.cn(Y.Z.)

This PDF file includes:

Supplementary Figures 1-13

Supplementary Tables 1

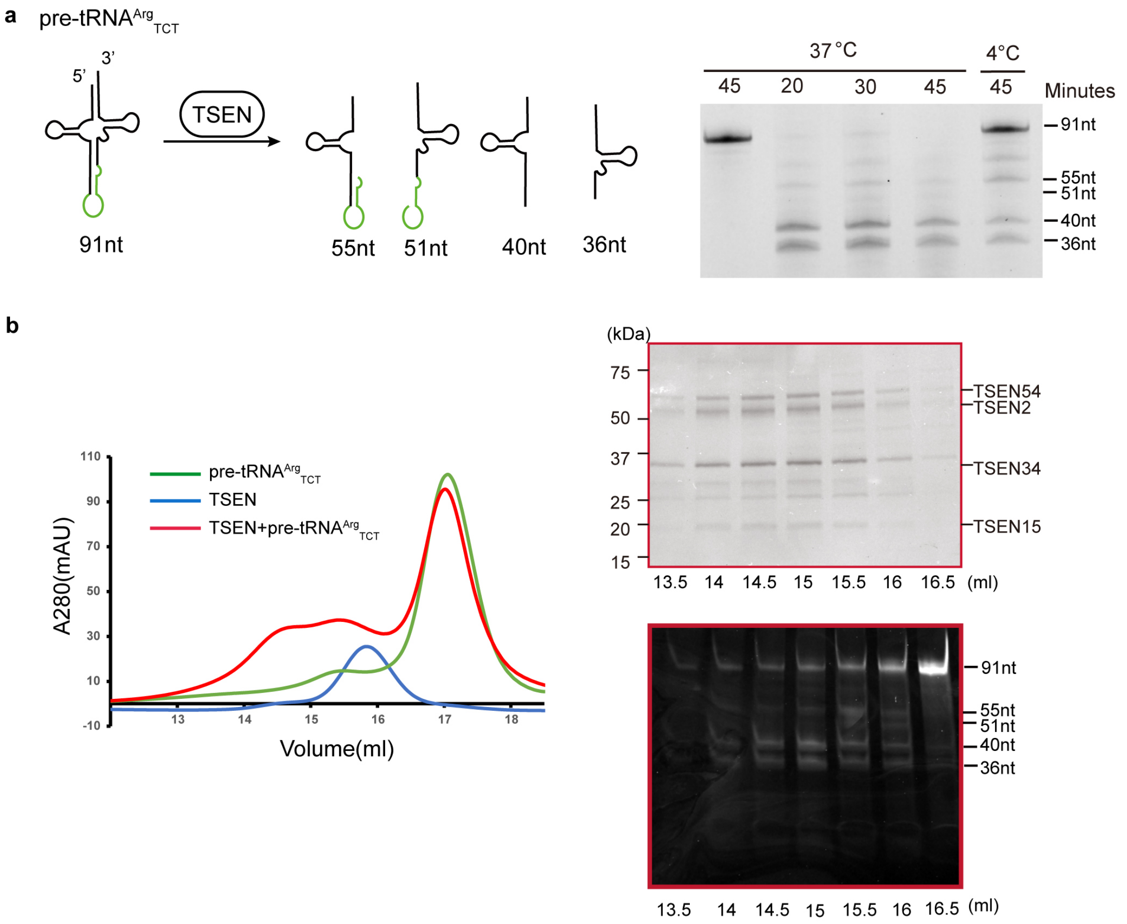

Supplementary Figure 1. **Reconstitution of an active human tRNA splicing machinery**. **a** Cleavage activity assay of a human pre-tRNA^{Arg} substrate by human TSEN complex in a 1:1 RNA : protein ratio. The mixture was incubated at 37 ℃ or 4 ℃ for different time and analyzed using a 15% denaturing Urea-PAGE gel, which was subsequently stained with 4SGelred. **b** Gel filtration profiles of the TSEN complex (blue), the pre-tRNA (green), and a mixture of TSEN and pre-tRNA (red). The elution volume of TSEN shifted from 15.8 ml to 14.6 ml in the presence of pre-tRNA. Top panel on the right: SDS-PAGE gel of the peak fractions. Bottom panel on the right: 15% denaturing Urea-PAGE gel of the peak fractions. The pre-tRNA used in this assay was the same as that in panel a. Source data are provided as a Source Data file.

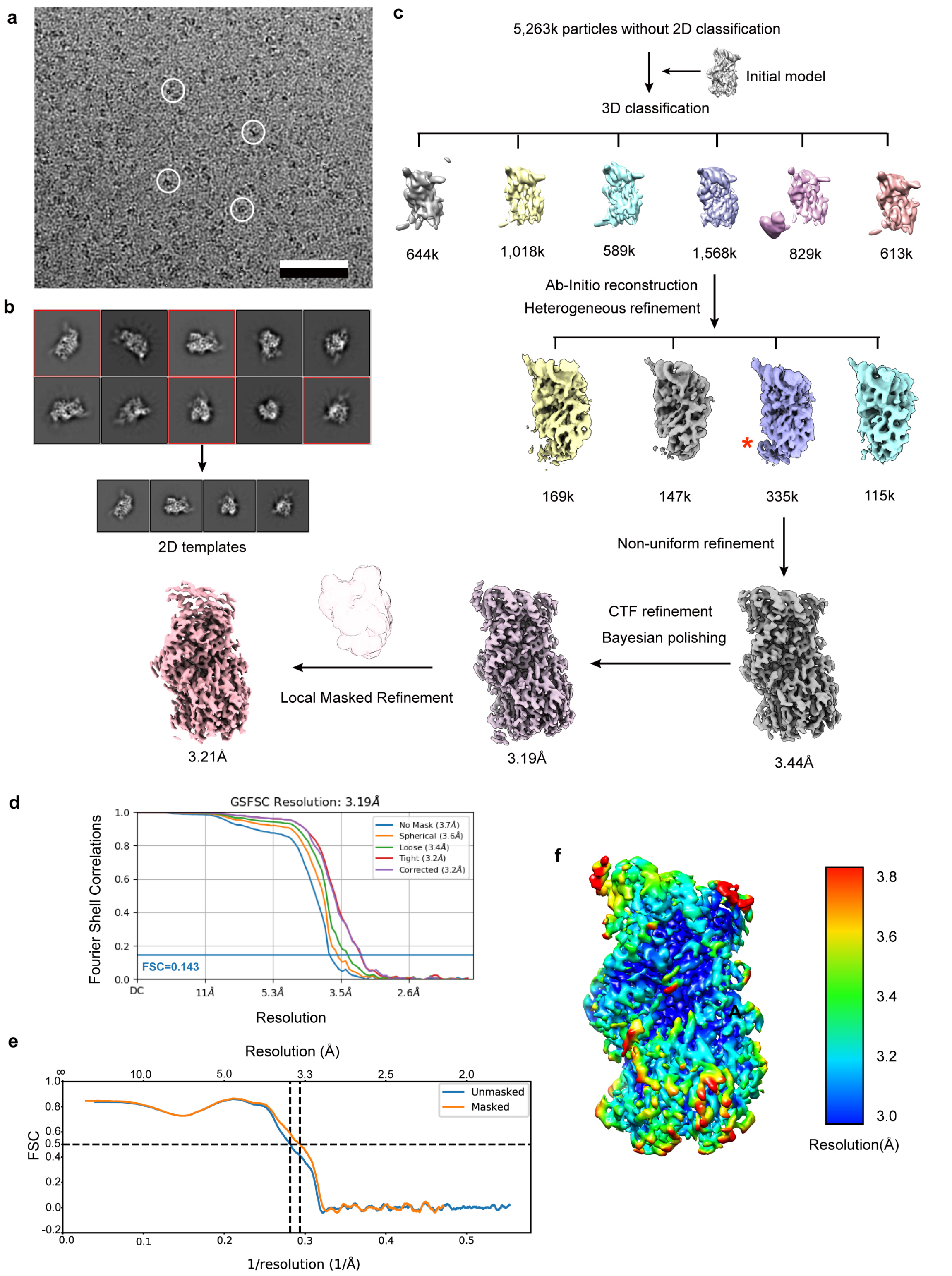

Supplementary Figure 2. **Cryo-EM image processing of the TSEN–pre-tRNA complex and quality assessment of the density maps. a** Area of a cryo-EM image of the vitrified machinery. Some particles are circled. Scale bar: 50 nm. **b** Selected 2D class averages obtained with RELION-3. **c** Image-processing workflow that resulted in a density map at

3.19 Å resolution. See Methods for details. **d** Gold-Standard Fourier Shell Correlations (FSC) curves of the full machinery. The gold-standard cutoff (FSC = 0.143) is marked with a blue line. **e** Model-to-map FSC curves. **f** Local resolution map for the TSEN–pre-tRNA complex.

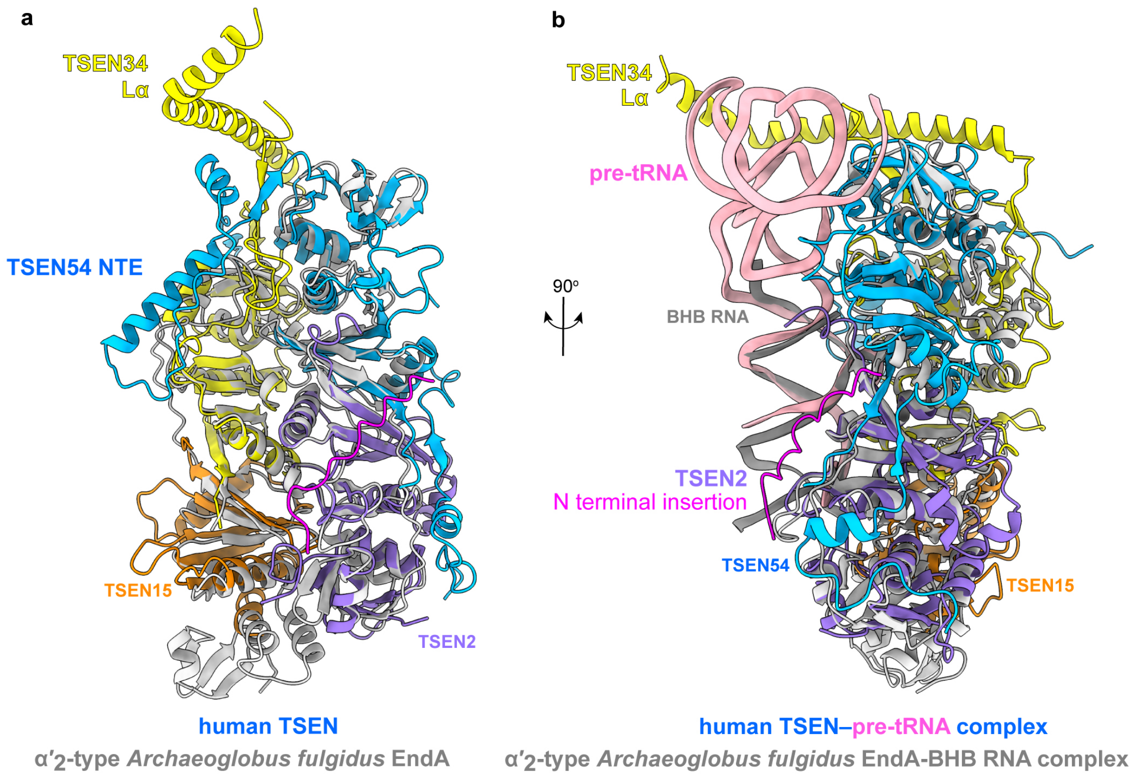

Supplementary Figure 3. **Comparison of human tRNA splicing machinery with the archaeal RNA-splicing endonucleases. a** Overlay of the structure of human TSEN reported here (in color) with that of α′$_2$-type *Archaeoglobus fulgidus* EndA (light gray, PDB entry 2GJW). The subunits of TSEN are given different colors. The unique insertions of eukaryotic subunits are labelled. **b** Same as panel a, viewed after a 90° rotation around the vertical axis. The tRNA substrates are shown to compare the similar locations of their anticodon domains on the tRNA splicing machinery.

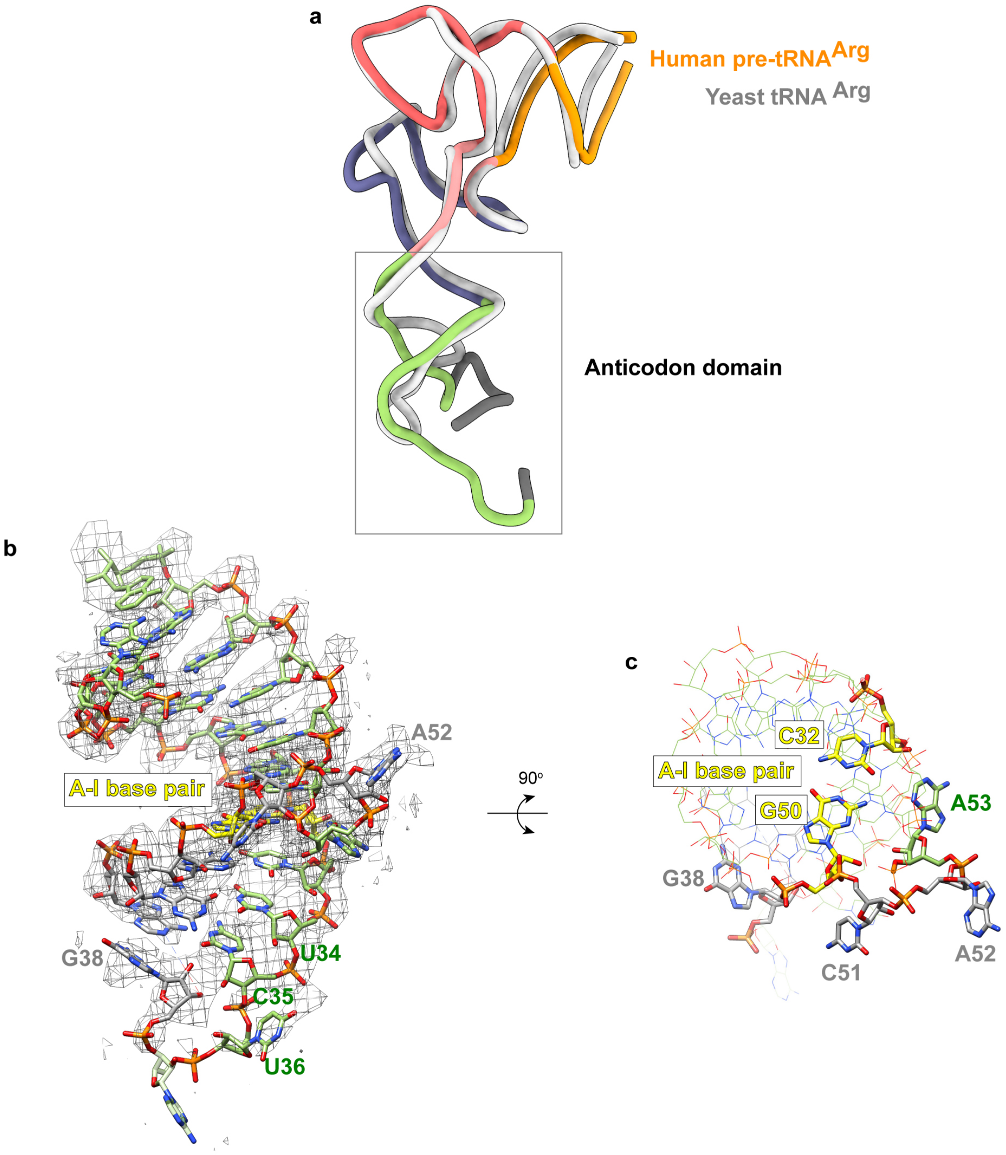

Supplementary Figure 4. **The three-dimensional structure of pre-tRNA. a** Overlay of the structure of human pre-tRNA^{Arg} with that of yeast tRNA^{Arg}. The RNA molecules are shown as cartoons, with human pre-tRNA (this work) in the same color scheme as in Fig.1c and yeast tRNA (PDB entry 1F7V) in light gray. The anticodon domain is indicated in the gray box. **b** EM density for the anticodon domain. The RNA is shown as sticks. A-I base pair: yellow; intron: gray. The A-I stem stacks on the anticodon stem to form a coaxial helix. The locations of anticodon (U34, C35, and U36), G38, and A52 are indicated. The density for scissile phosphate and G38 is clear. **c** Same as panel b, viewed after a 90° rotation around the horizontal axis. The locations of the A-I base pair and 3' bugle are highlighted as sticks, while other nucleotides in the anticodon domain are shown as wires. The three flipped-out bases, C51, A52, and A53, are labelled.

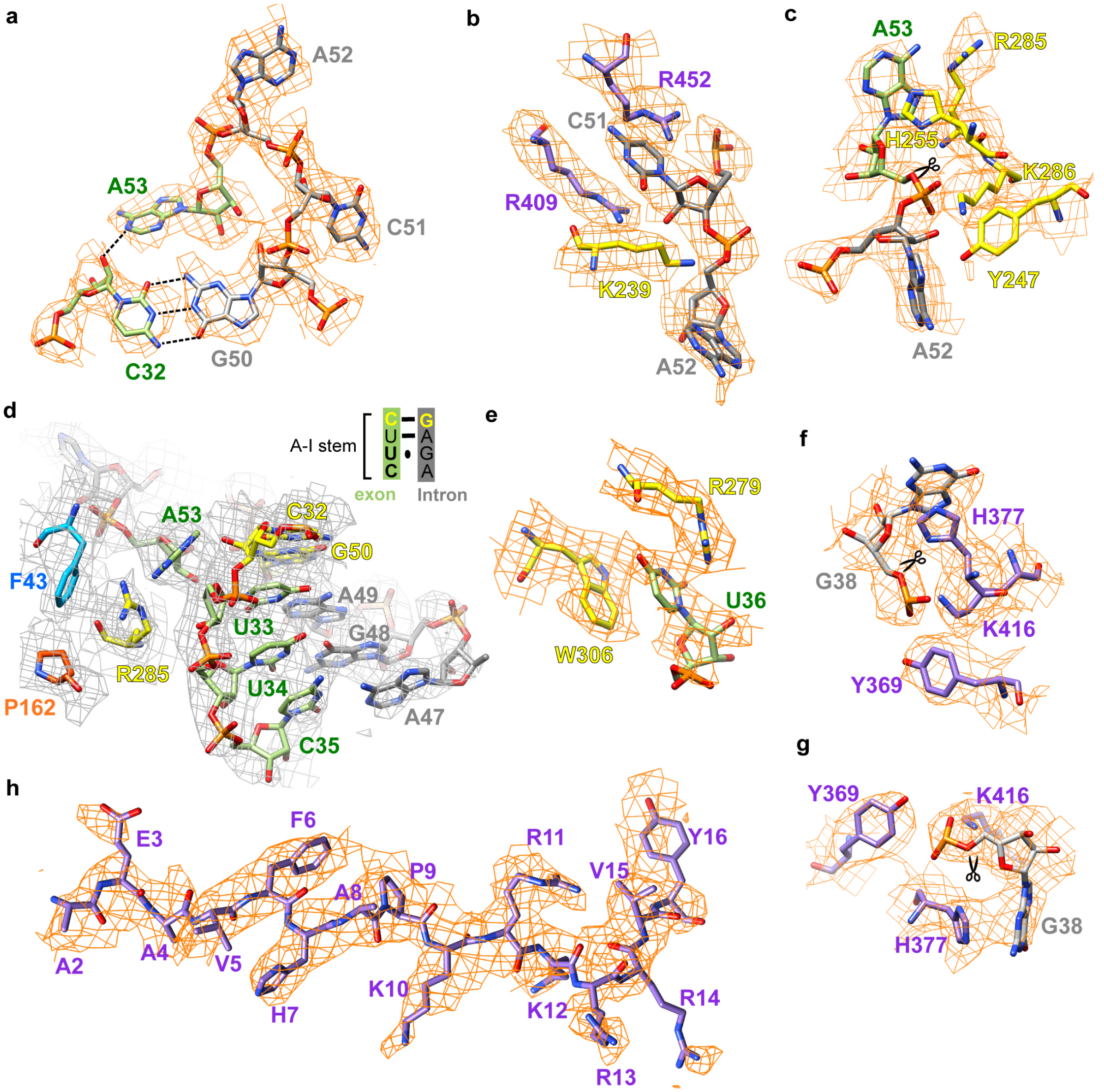

Supplementary Figure 5. **Representative cryo-EM densities of the two splice boundaries and A-I stem by human TSEN complex. Related to Figures 3b, 4b, 5a, and Supplementary Figs. 4b-c. a** The EM map for the 3' bulge and A-I base pair. The last bulge nucleotide, A53, forms hydrogen-bonding interaction with the A-I base pair. The scissile phosphate, indicated with the symbol 'scissors'. **b** The EM map for the interface surrounding C51. **c** The EM map at the interface between 3' splice site and the catalytic triad of TSEN34. **d** A close-up view of the EM map focusing on the interface near the A-I stem. The density surrounding A-I stem exhibits good quality, and there was no indication of any density being pulled out or disconnected from the A-I stem. **e** The EM map for the R279-W306 tweezer in TSEN34. **f** and **g** The EM map at the interface between 5' splice site and the catalytic triad of TSEN2. **h** The EM map of the NTE of TSEN2.

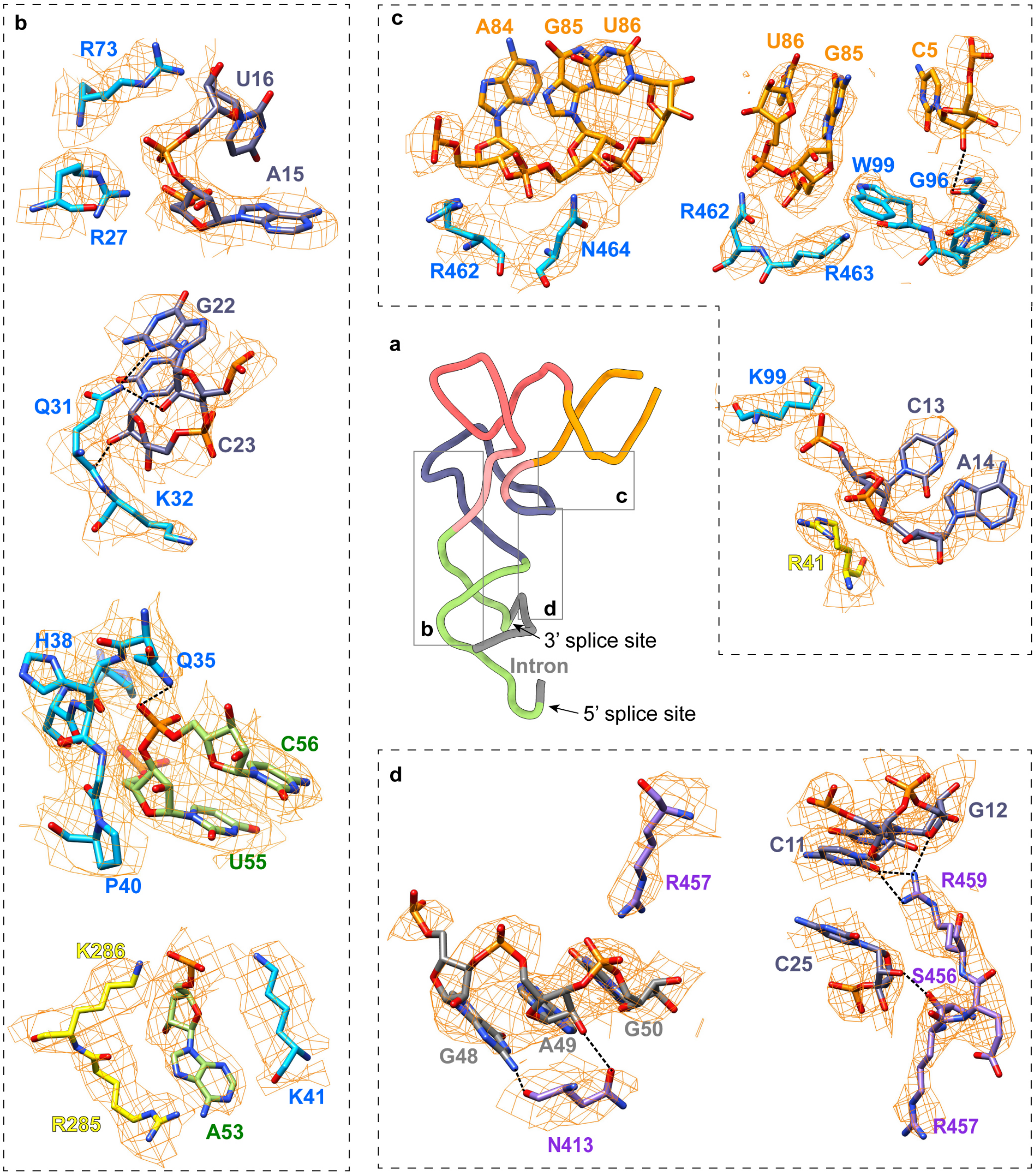

Supplementary Figure 6. **The local EM maps of the interface between human TSEN and pre-tRNA. Related to Figures 3b, 3c, and 3f. a** The 3D structure of pre-tRNA^{Arg}. The color scheme is the same as in Fig. 1c. **b, c and d** Close-up views depicting the interactions between pre-tRNA and proteins. **b** Detail views on the EM maps of D arm and anticodon stem recognition by the NTE of TSEN54. Key residues that are involved in recognition are shown as sticks. **c** Close-up views on the EM maps of the acceptor stem and TSEN54. R41 from P-loop of TSEN34 is also indicated. **f** Close-up views on the EM maps of interfaces between TSEN2 and pre-tRNA.

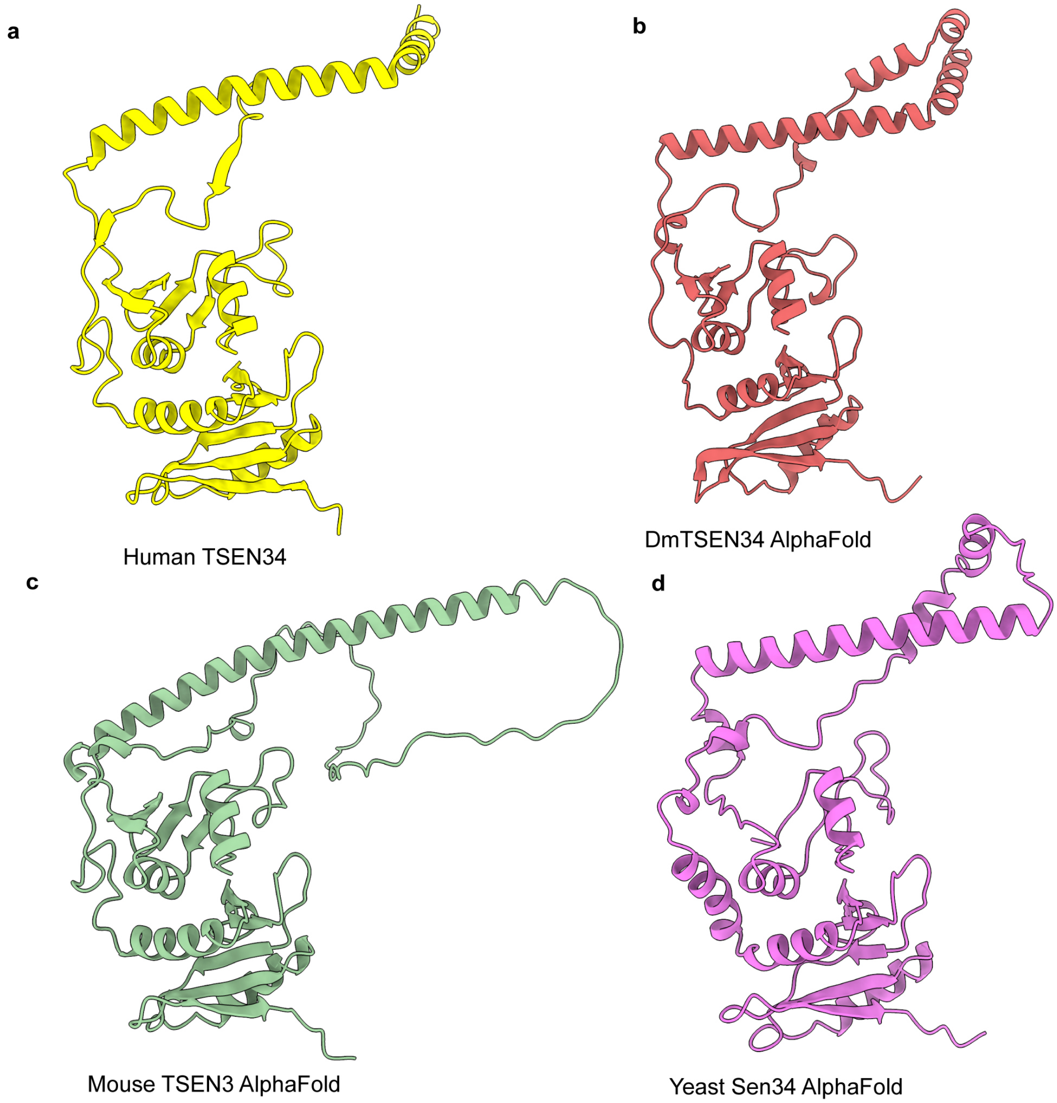

Supplementary Figure 7. **AlphaFold or cryo-EM structures of TSEN34 from different species.** Comparison of the architecture of human TSEN (a, this study) and AlphaFold models of *Drosophila melanogaster* TSEN34 (b), mouse TSEN34 (c), and yeast Sen34 (d).

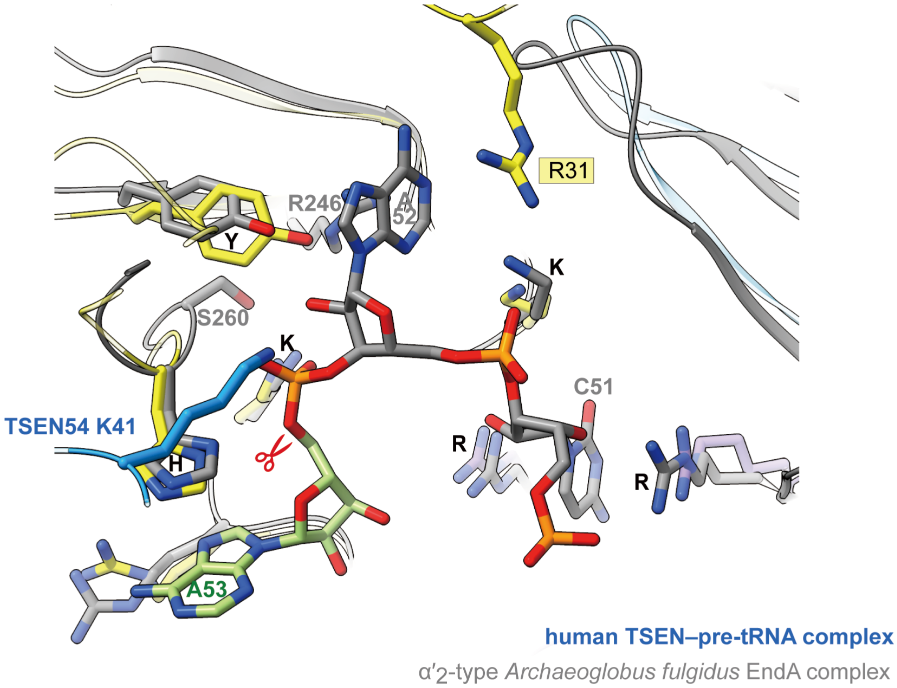

Supplementary Figure 8. **Detailed comparison of the catalytic center in human tRNA splicing machinery and the archaeal RNA-splicing endonucleases.** Overlay of the structure of human TSEN reported here (colored as in Fig. 1a) with that of α′$_2$-type *Archaeoglobus fulgidus* EndA (light gray, PDB entry 2GJW). The cleavage site is indicated with the symbol 'scissors', and the 3' bugle nucleotides of human pre-tRNA are displayed. The binding modes of human TSEN for the 3' bugle overlap with those of archaeal EndA, indicating the conserved recognition and catalytic mechanism for the removal of tRNA introns in archaeal and eukaryotic endonucleases. R31 of TSEN34 and K41 of TSEN54 are additional contacts in human TSEN.

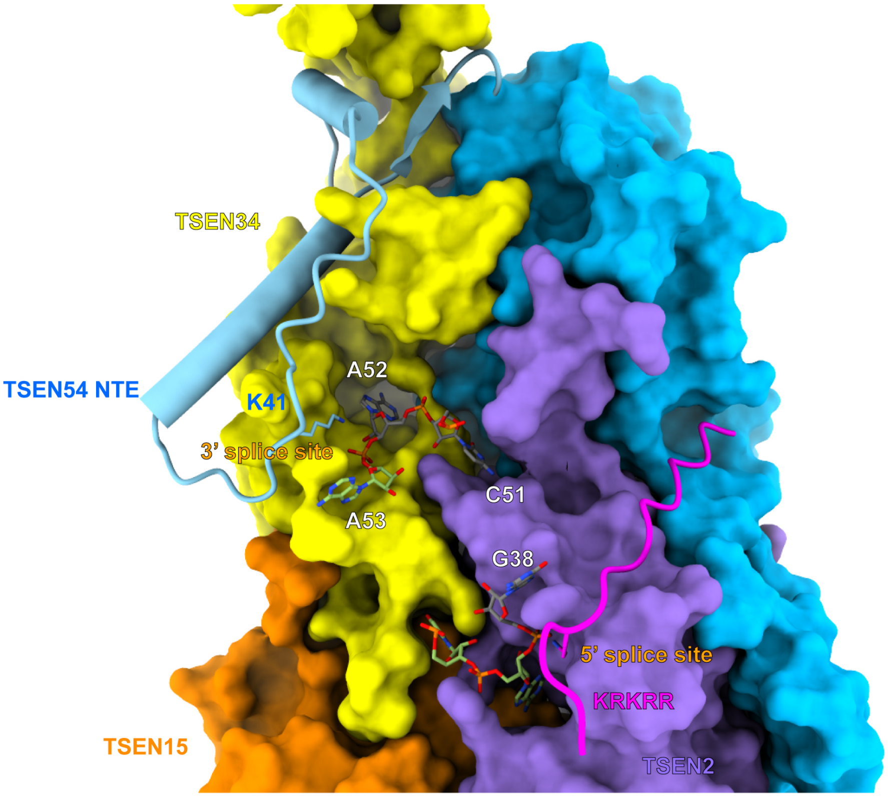

Supplementary Figure 9. **Two insertion loops from TSEN54 and TSEN2 cover the 3’ and 5’ splice sites, respectively.** Molecular surface of the proteins in human TSEN complex, colored as in Fig. 1a. The N-terminal insertions of TSEN54 (residues 1-75) and TSEN2 (residues 1-16) are shown as cartoons. The 3’ and 5’ splice boundaries of pre-tRNA are shown as sticks.

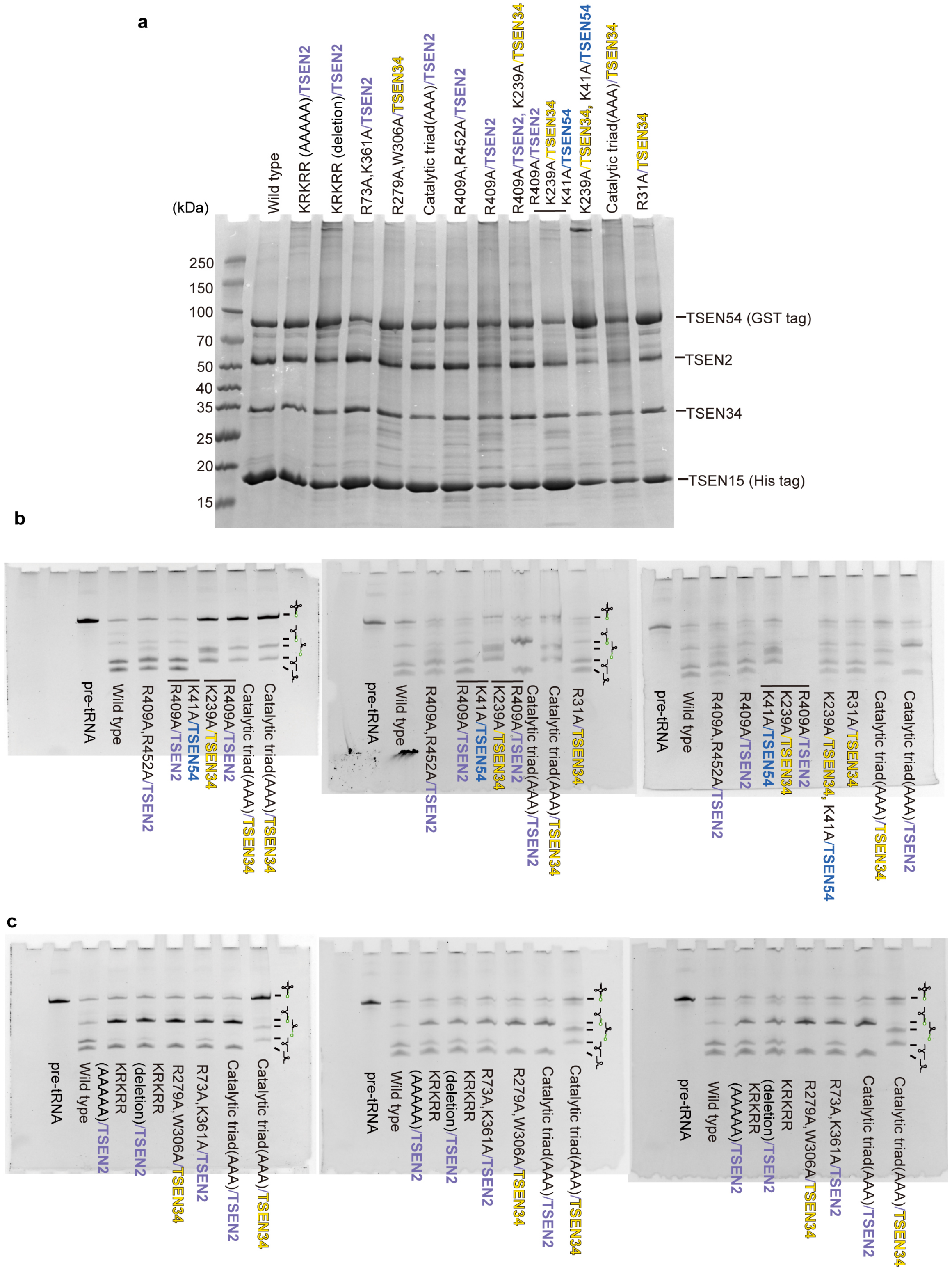

Supplementary Figure 10. **Cleavage assays to assess the impact of various mutations on the cleavage activity at the 3’ and 5' splice site. a** A SDS-PAGE gel of all enzymes used for the assay. The His-tagged protein was extracted from a 50ml culture of Hi5 cells. **b** and

c Related to Figs. 4c and 5d. The cleavage assays were repeated three times. Source data are provided as a Source Data file.

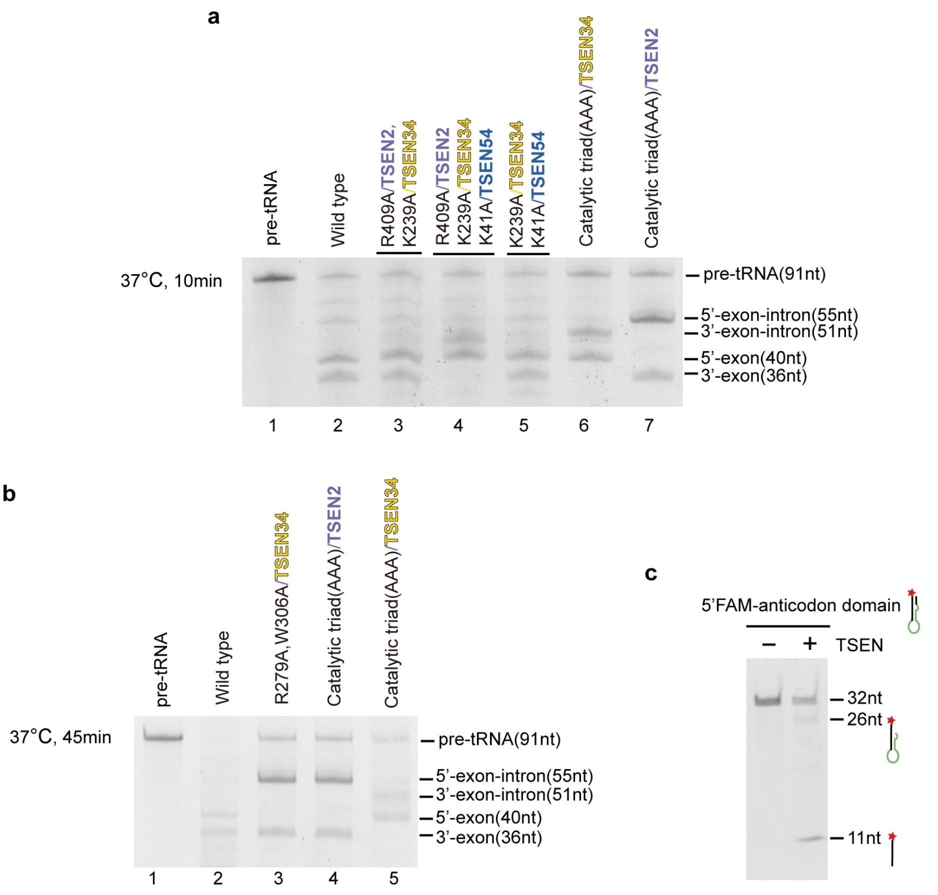

Supplementary Figure 11. **Cleavage assays to assess the impact of various mutations on the cleavage activity.** **a** Cleavage assays showing that introducing the K41A mutation in TSEN54 or the R409A mutation in TSEN2 enhances the effect of the double mutants formed by R409^{TSEN2}/K239^{TSEN34} (lane 3) or K41^{TSEN54}/K239^{TSEN34} (lane 5), resulting in a complete loss of cleavage (lane 4). **b** Cleavage assays to assess the impact of the mutation of the R279-W306 tweezer in TSEN34. The reaction time is 10min in Fig. 5d. To rule out the possibility of insufficient reaction time as a factor, we repeated this assay with a longer reaction time of 45 min. Notably, even with the longer reaction time, the mutation of the R279-W306 tweezer in TSEN34 still led to the complete abolishment of cleavage at the 5' splice site (lane 3). **c** Cleavage of a pre-tRNA mimic by the human TSEN complex. The pre-tRNA mimic only contains the anticodon domain with a Bulge-Helix-Loop motif. The intron part is indicated in green. The 5' products with a FAM label are detected on the 15% denaturing Urea-PAGE gel. Source data are provided as a Source Data file.

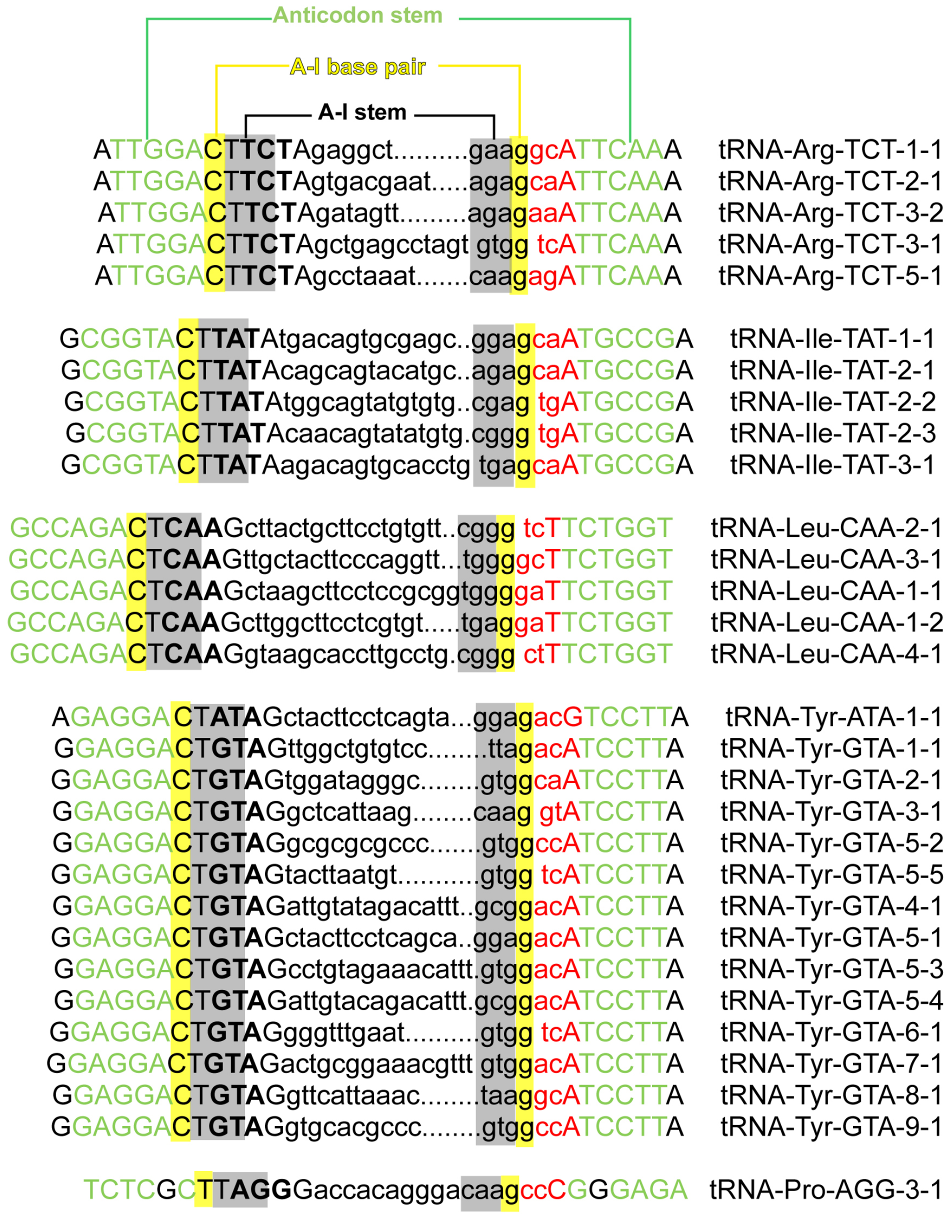

Supplementary Figure 12. **The conservation of the anticodon-intron (A-I) stem and 3' bulge structures in human pre-tRNAs.** Structural alignments of intron-containing pre-tRNAs from *Homo sapiens*. The sequences of intron-containing pre-tRNAs are downloaded from GtRNAdb. Only the anticodon domains are shown here. The introns are shown in lowercase. The anticodon stem is in green and the 3' bulge in red, while the predicted A-I stem is in gray, with the A-I base pair highlighted in yellow. Except for $tRNA^{Tyr}_{GTA}$ 3-1 and $tRNA^{Tyr}_{GTA}$ 8-1, at least three base pairs are formed in the A-I stem, including both the Watson-Crick and the G・U wobble base pairing.

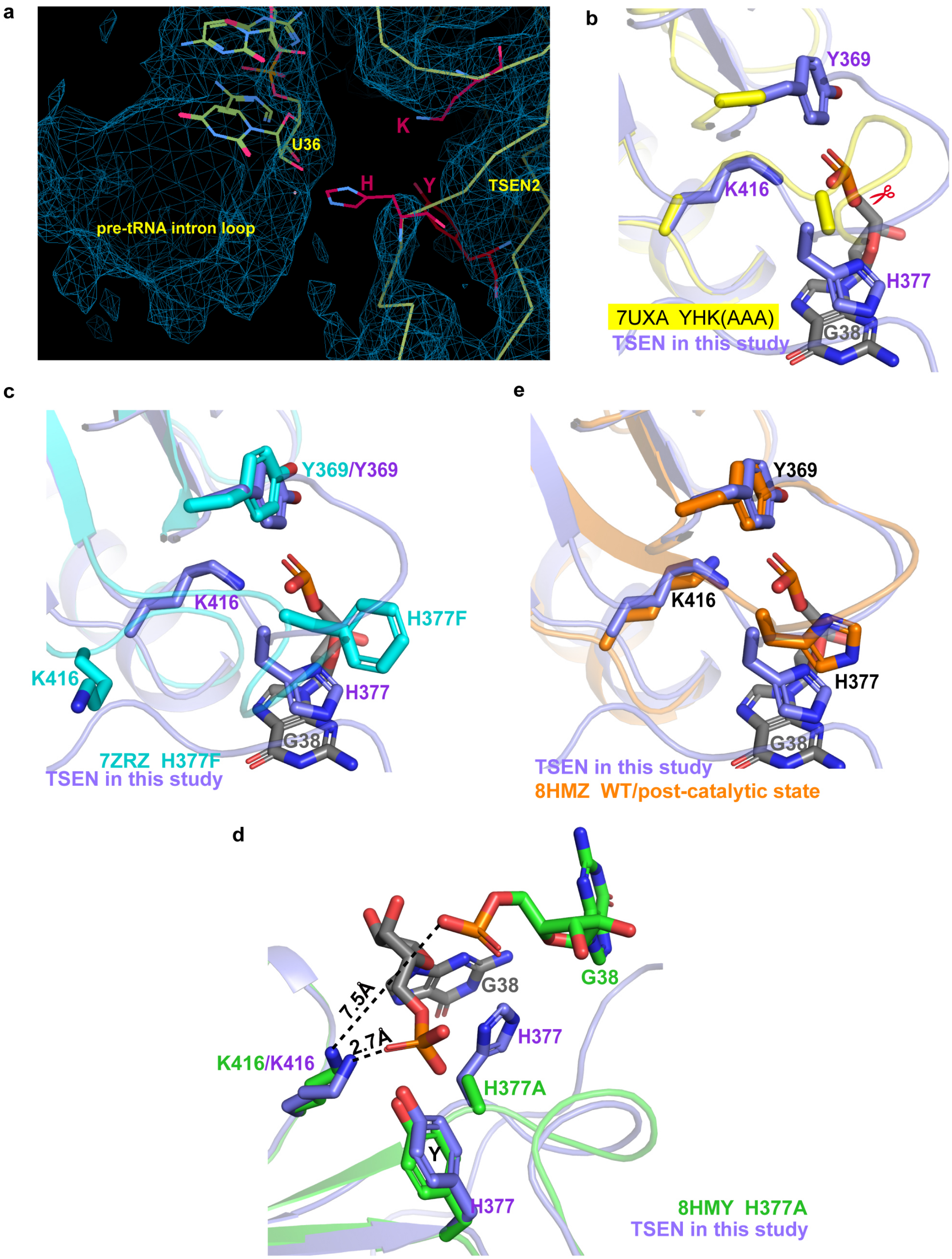

Supplementary Figure 13. **Detailed comparison of the catalytic center of the 5' splice site in this study with those in other states. a** The density map for the TSEN–pre-tRNA complex with the mutations of all three catalytic residues to Ala in TSEN2 (EMD-26856). The positions of U36 and the regions of pre-tRNA intron loop and TSEN2 subunit are labeled (PDB entry 7UXA). The potential positions of the catalytic triad are indicated as red sticks. Based on the density map, no RNA density was observed in the binding pocket formed by the catalytic triad. **b** Overlay of the structure of human TSEN reported here

(colored as in Fig. 1a) with that with triple mutants, Y369A, H377A, and K416A, in TSEN2 (yellow, PDB entry 7UXA, related to panel a). G38 is shown as a reference to indicate the active site. The cleavage site is indicated with the symbol ‘scissors’. **c** Overlay of the structure of human TSEN reported here (colored as in Fig. 1a) with that of the H377F mutant in TSEN2 (cyan, PDB entry 7ZRZ). The crucial residue K416 in 7ZRZ exhibited a different conformation, being distant from the active site. **d** Overlay of the structure of human TSEN reported here (colored as in Fig. 1a) with that of the H377A mutant in TSEN2 (green, PDB entry 8HMY). Similarly, in the TSEN complex with TSEN2 H377A mutant, the distance between the active site and the scissile phosphate of G38 is too far. **e** Overlay of the structure of human TSEN reported here (colored as in Fig. 1a) with that of wild-type TSEN in post-catalytic state (orange, PDB entry 8HMZ). For the structure of wild-type TSEN in the post-catalytic state, the sample revealed almost complete cleavage of the products, resulting in the absence of density for the scissile phosphate.

Supplementary Table 1

Cryo-EM data collection, refinement and validation statistics

	Human TSEN–pre-RNA^{Arg} complex (EMD-35694) (PDB 8ISS)
**Data collection and processing**	
Magnification	81,000
Voltage (kV)	300
Electron exposure ($e^{–}/Å^2$)	49.4
Defocus range (μm)	1.4 to 2.4
Pixel size (Å)	1.055
Symmetry imposed	C1
Image stacks (no.)	4500
Initial particle images (no.)	5,263,873
Final particle images (no.)	335,714
Map resolution (Å)	3.19
FSC threshold	0.143
Map sharpening B-factor ($Å^2$)	-159.6
**Refinement**	
Number of protein residues	889
Number of RNA nucleotides	80
Number of metal ions	1
B-factor (min/max/mean)	
Protein	0.00/137.27/60.91
Nucleotide	15.61/17817.32/483.95
CC (mask)	0.75
Main chain CC; side chain CC	0.77; 0.71
R.m.s. deviations	
Bond lengths (Å)	0.002
Bond angles (°)	0.514
PDB validation	
Clash score	15
Poor rotamers (%)	0.52
Ramachandran plot	
Favored (%)	94.16
Allowed (%)	5.84
Disallowed (%)	0.00
