# Supplementary material for: Recognition and cleavage mechanism of intron-containing pre-tRNA by human TSEN endonuclease complex
Source: Nat Commun. 2023 Sep 28;14:6071. doi: 10.1038/s41467-023-41845-y (PMC10539383; doi:10.1038/s41467-023-41845-y)
Supplement: Supplementary file 3 — Description of Additional Supplementary Files [file 41467_2023_41845_MOESM3_ESM.pdf]

Description of Additional Supplementary Files

File Name: Supplementary Data 1
Description: to list the sequences of oligonucleotides (e.g. primers and RNAs)
