# Supplementary material for: Recognition and cleavage mechanism of intron-containing pre-tRNA by human TSEN endonuclease complex
Source: Nat Commun. 2023 Sep 28;14:6071. doi: 10.1038/s41467-023-41845-y (PMC10539383; doi:10.1038/s41467-023-41845-y)
Supplement: Supplementary file 5 — Reporting Summary [file 41467_2023_41845_MOESM5_ESM.pdf]

nature portfolio

Corresponding author(s): Yadong Sun,Yixiao Zhang

Last updated by author(s): Sep 5, 2023

Reporting Summary

Nature Portfolio wishes to improve the reproducibility of the work that we publish. This form provides structure for consistency and transparency in reporting. For further information on Nature Portfolio policies, see our Editorial Policies and the Editorial Policy Checklist.

Statistics

For all statistical analyses, confirm that the following items are present in the figure legend, table legend, main text, or Methods section.

n/a	Confirmed	
☐	☒	The exact sample size (n) for each experimental group/condition, given as a discrete number and unit of measurement
☐	☒	A statement on whether measurements were taken from distinct samples or whether the same sample was measured repeatedly
☒	☐	The statistical test(s) used AND whether they are one- or two-sided<br>*Only common tests should be described solely by name; describe more complex techniques in the Methods section.*
☒	☐	A description of all covariates tested
☒	☐	A description of any assumptions or corrections, such as tests of normality and adjustment for multiple comparisons
☐	☒	A full description of the statistical parameters including central tendency (e.g. means) or other basic estimates (e.g. regression coefficient) AND variation (e.g. standard deviation) or associated estimates of uncertainty (e.g. confidence intervals)
☒	☐	For null hypothesis testing, the test statistic (e.g. F, t, r) with confidence intervals, effect sizes, degrees of freedom and P value noted<br>*Give P values as exact values whenever suitable.*
☒	☐	For Bayesian analysis, information on the choice of priors and Markov chain Monte Carlo settings
☒	☐	For hierarchical and complex designs, identification of the appropriate level for tests and full reporting of outcomes
☒	☐	Estimates of effect sizes (e.g. Cohen's d, Pearson's r), indicating how they were calculated

Our web collection on statistics for biologists contains articles on many of the points above.

Software and code

Policy information about availability of computer code

Data collection	cryo-EM data were collected with EPU
Data analysis	MotionCor2 v1.4.4, cryoSPARC V4.0, CTFFIND4, Relion V3.1, Coot v0.8.8, Phenix v1.17.1, UCSF ChimeraX v1.5, UCSF Chimera 1.16, PyMOL 2.5.4

For manuscripts utilizing custom algorithms or software that are central to the research but not yet described in published literature, software must be made available to editors and reviewers. We strongly encourage code deposition in a community repository (e.g. GitHub). See the Nature Portfolio guidelines for submitting code & software for further information.

Data

Policy information about availability of data

All manuscripts must include a data availability statement. This statement should provide the following information, where applicable:

- Accession codes, unique identifiers, or web links for publicly available datasets
- A description of any restrictions on data availability
- For clinical datasets or third party data, please ensure that the statement adheres to our policy

The following publicly available data were used in the manuscript: for tRNA model building, PDB coordinates of PDB-ID 2GJW and 1F7V; for structural comparison, EMD-26856 and PDB coordinates of PDB-ID 7UXA, 7ZRZ, 8HMZ and 8HMY.

The atomic coordinates and the EM maps in the paper are available in the Protein Data Bank (PDB) at http://www.pdb.org. Accession codes are PDB 8ISS and EMDB-35694.

Research involving human participants, their data, or biological material

Policy information about studies with human participants or human data. See also policy information about sex, gender (identity/presentation), and sexual orientation and race, ethnicity and racism.

Reporting on sex and gender	none
Reporting on race, ethnicity, or other socially relevant groupings	none
Population characteristics	none
Recruitment	none
Ethics oversight	none

Note that full information on the approval of the study protocol must also be provided in the manuscript.

Field-specific reporting

Please select the one below that is the best fit for your research. If you are not sure, read the appropriate sections before making your selection.

☒ Life sciences ☐ Behavioural & social sciences ☐ Ecological, evolutionary & environmental sciences

For a reference copy of the document with all sections, see nature.com/documents/nr-reporting-summary-flat.pdf

Life sciences study design

All studies must disclose on these points even when the disclosure is negative.

Sample size	Sample sizes for the Cryo-EM datasets were determined by the need to obtain meaningful structures, which was determined by the number of extractable particles from the movie stacks for further image processing.<br>For human TSEN–pre-tRNA, 4500 movie stacks were collected and 335,714 particles were used for the final reconstruction, which was sufficient to yield a 3.19-Å resolution structure.
Data exclusions	Micrographs clearly suffering from astigmatism, image drift, and ice contamination were excluded from the datasets. Particles in 3D classes showing poor structural features were excluded from the final reconstructions.
Replication	A small dataset was first collected and a medium resolution map was confirmed by this small dataset. For the high-resolution structure obtained in this study, we did a large data collection on the 300-kV Titan Krios electron microscope. The replication were successful. The resolution of the structure was improved to 3.19-Å .<br>The cleavage assay of TSEN mutations were repeated three times. All attempts at replication were successful.
Randomization	In the 3D refinement, particle images were randomly split into two half groups.
Blinding	Blinding is not applicable for this study, as group allocation is not used. All experiments were performed in vitro whereby the investigator both designs and performs said experiment.

Reporting for specific materials, systems and methods

We require information from authors about some types of materials, experimental systems and methods used in many studies. Here, indicate whether each material, system or method listed is relevant to your study. If you are not sure if a list item applies to your research, read the appropriate section before selecting a response.

Materials & experimental systems

n/a	Involved in the study
☒	☐ Antibodies
☐	☒ Eukaryotic cell lines
☒	☐ Palaeontology and archaeology
☒	☐ Animals and other organisms
☒	☐ Clinical data
☒	☐ Dual use research of concern
☒	☐ Plants

Methods

n/a	Involved in the study
☒	☐ ChIP-seq
☒	☐ Flow cytometry
☒	☐ MRI-based neuroimaging

Eukaryotic cell lines

Policy information about cell lines and Sex and Gender in Research

Cell line source(s)	sf9 and High Five/Trichoplusia ni cells (insect cell line) were obtained from Expression Systems.
Authentication	No
Mycoplasma contamination	The cell lines were not tested for Mycoplasma contamination.
Commonly misidentified lines (See ICLAC register)	No
