# Supplementary material for: Recognition and cleavage mechanism of intron-containing pre-tRNA by human TSEN endonuclease complex
Source: Nat Commun. 2023 Sep 28;14:6071. doi: 10.1038/s41467-023-41845-y (PMC10539383; doi:10.1038/s41467-023-41845-y)

**Figure 4c** Cleavage assays

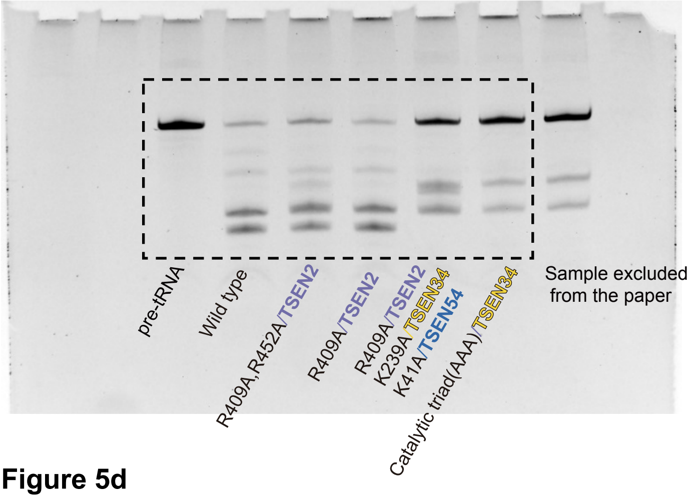


**Figure 5d**

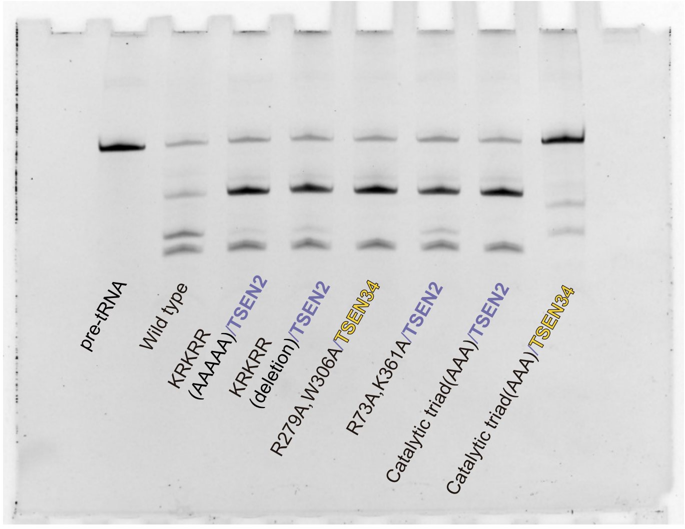


**Supplementary Figure 1a**

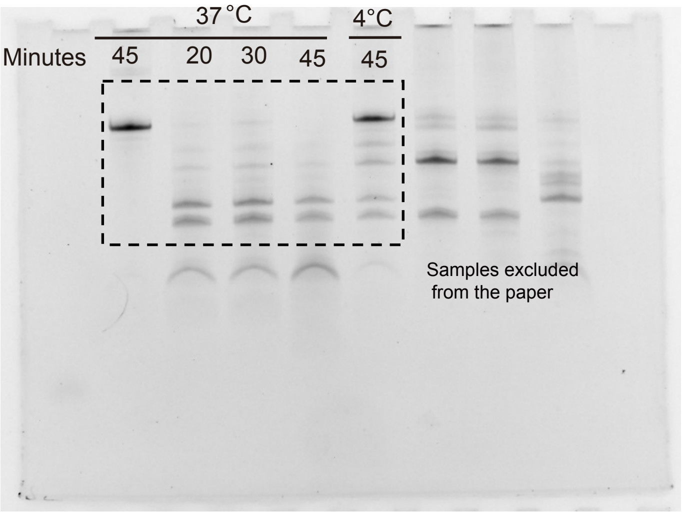

## Supplementary Figure 1b

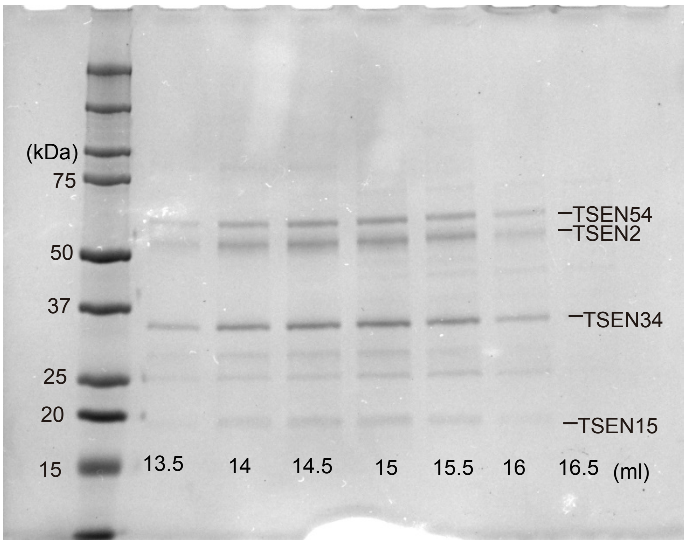


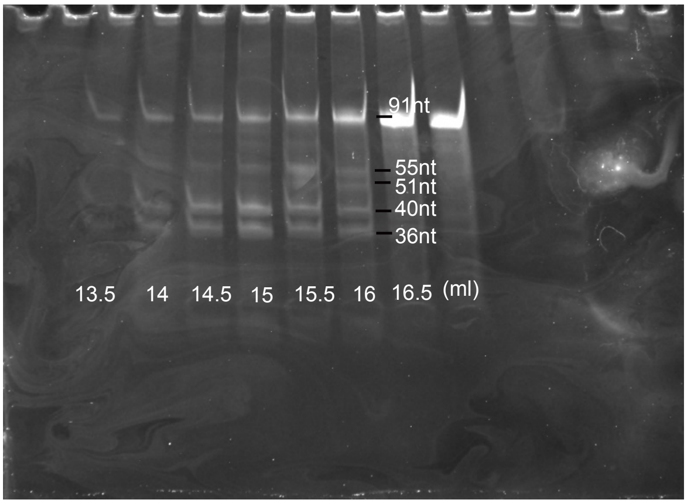


## Supplementary Figure 10a

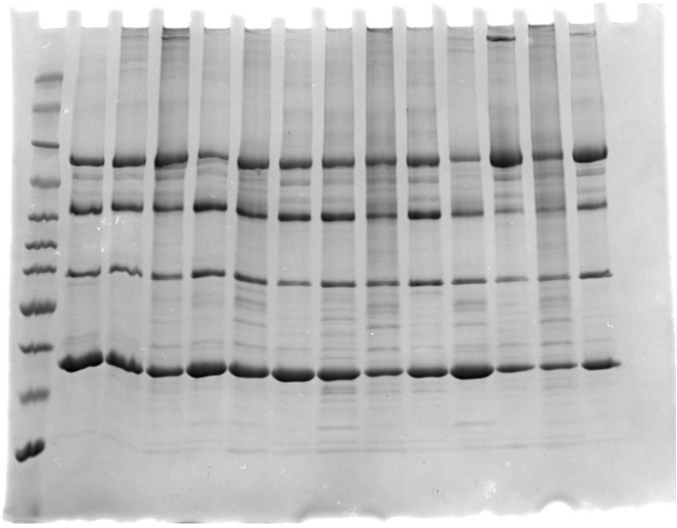

**Supplementary Figure 10b**

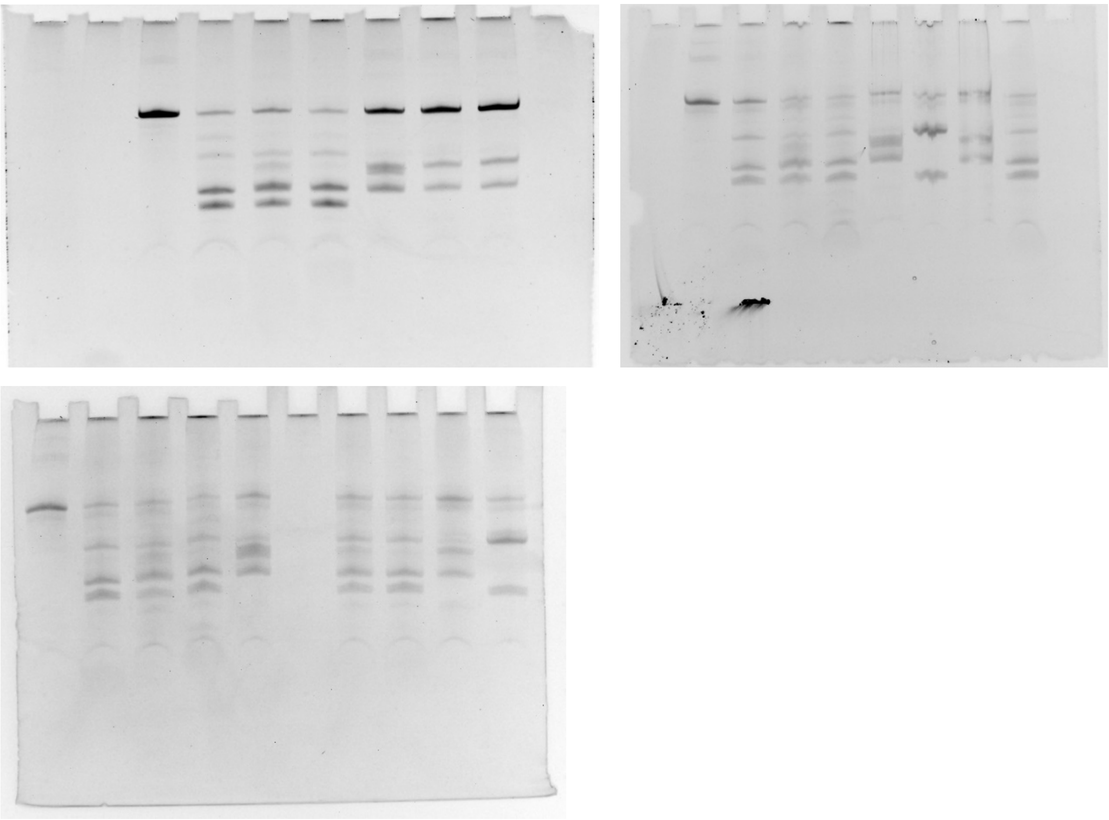

**Supplementary Figure 10c**

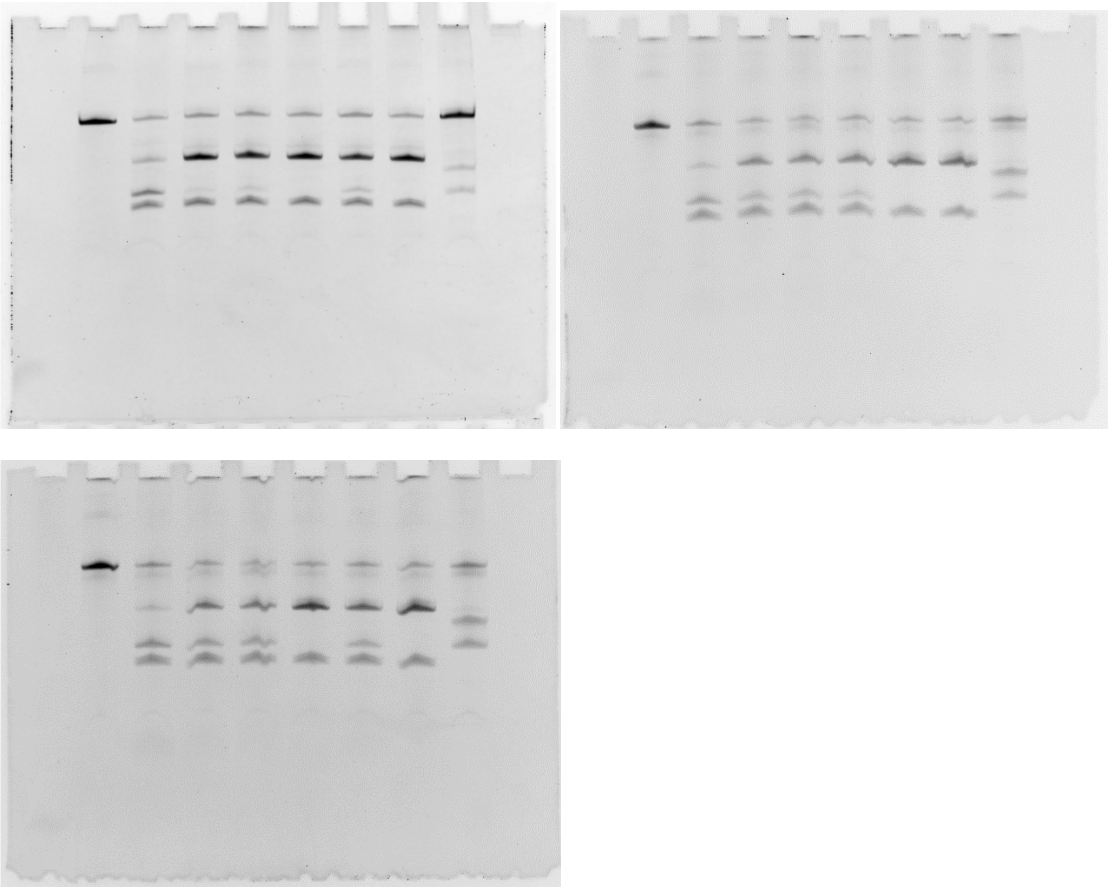

## Supplementary Figure 11a

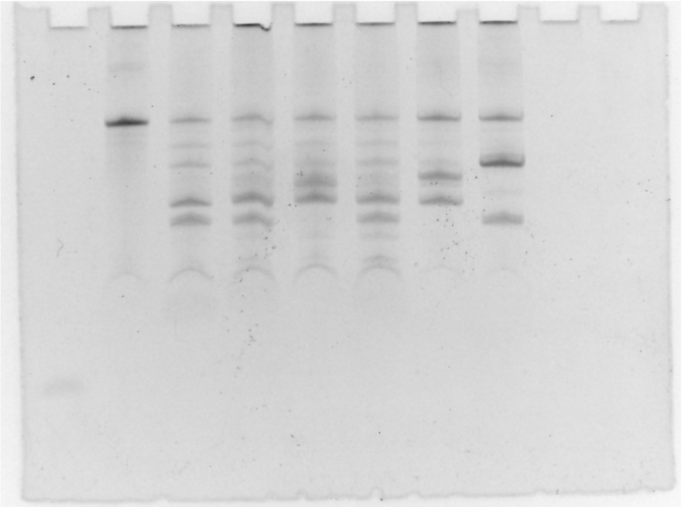

## Supplementary Figure 11b

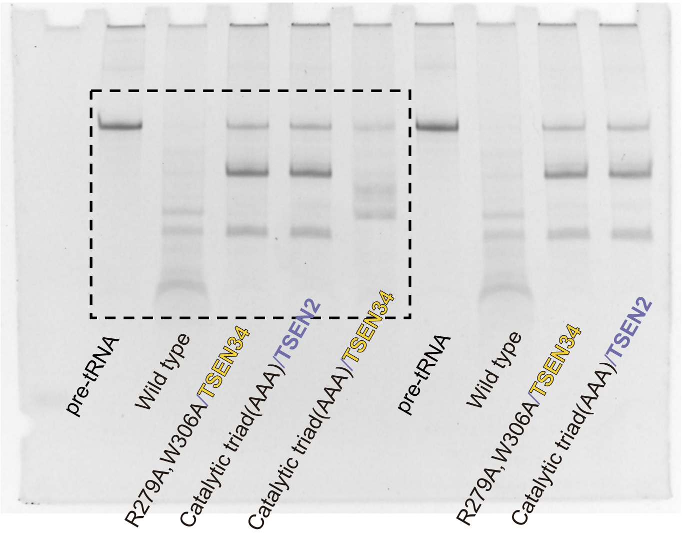


## Supplementary Figure 11c

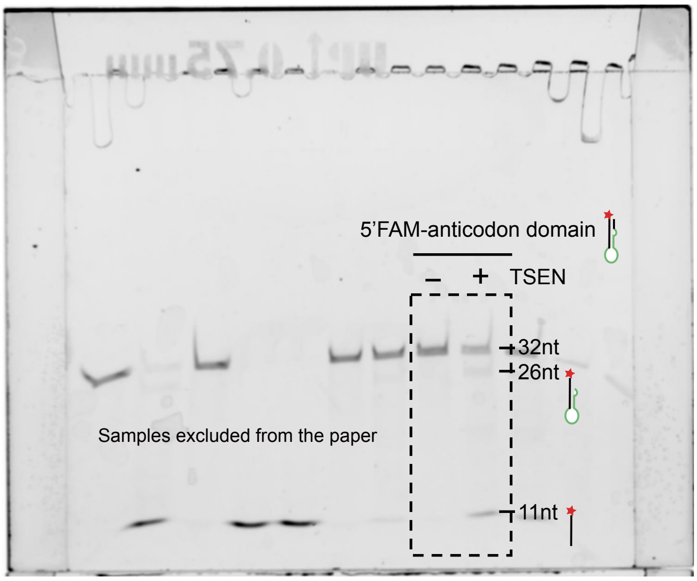

Supplement: Supplementary file 6 — Source Data [file 41467_2023_41845_MOESM6_ESM.zip › Source Data.pdf]
